# New Damage Accumulation Model for Spall Propagation Mechanism in Bearing Raceways

**DOI:** 10.3390/ma16041750

**Published:** 2023-02-20

**Authors:** Ravit Ohana, Renata Klein, Roni Shneck, Jacob Bortman

**Affiliations:** 1 PHM Laboratory, Department of Mechanical Engineering, Ben-Gurion University of the Negev, P.O. Box 653, Beer-Sheva 8410501, Israel; 2 R. K. Diagnostics, Gilon, P.O. Box 101, D. N. Misgav 2010300, Israel; 3 Department of Material Engineering, Ben-Gurion University of the Negev, P.O. Box 653, Beer-Sheva 8410501, Israel

**Keywords:** rolling element bearings, spall propagation, damage mechanics, fatigue crack growth, finite element

## Abstract

The aim of this study was to investigate the spall propagation mechanism in ball bearing raceways using physics-based models. Spalling is one of the most common types of bearing failures that can lead to catastrophic failure. This research takes a step forward toward developing a prognostic tool for ball bearings. It is first necessary to understand the spall progression process in order to formulate a constitutive law of spall deterioration and to estimate the amount of remaining useful life. Fragment formation in the vicinity of the spall edge was found to consist of surface and sub-surface cracks that eventually coalesce, and a fragment is released from the raceway, based on naturally-developed spalls. Here, we describe a physics-based model, integrating a dynamic model with a finite element one to simulate this process. A continuum damage mechanics (CDM) approach and fracture mechanics tools were embedded into the finite element model to simulate the damage propagation. The formation of cracks in the vicinity of the spall (surface and sub-surface cracks) were studied using this effective stress CDM model, and the propagation of the cracks was examined using two approaches: a fracture mechanics approach and an accumulated inelastic hysteresis energy CDM approach. The latter also predicts the overall process of a single fragment release. The simulation results of the spall propagation models are supported by experimental results of spalls from both laboratory experimental bearings and an in-service Sikorsky CH-53 helicopter swashplate bearing. The results obtained show that the impact of the ball on the spall edge affects the crack propagation and the appearance of the surface and sub-surface cracks. Both release the residual stresses and cause crack propagation until a fragment is released.

## 1. Introduction (Spall Initiation and Propagation)

Understanding the driving mechanism of spall propagation is a step toward performing physics-based bearing prognostics for condition-based maintenance (CBM). These prognostics intend to enable the prediction of remaining useful life (RUL), and thereby improve safety and reduce costs and the probability of fault development. Spall evolution in raceways can be divided into three stages: (i) spall initiation by a rolling contact fatigue (RCF) mechanism, (ii) steady spall propagation, and (iii) accelerated spall propagation until final failure. As the spall initiation process has been studied both empirically [1,2,3] and theoretically [4,5,6,7,8,9], the present research focused on the spall propagation process and factors that affect it. While several studies attempted to quantify the spall growth mechanism, such as those of Arakere et al. [10] and Branch et al. [11,12], none have addressed the progression of cracks and removal of material from the spall edge, which results in flake release and spall propagation. Morales-Espejal et al. [13,14] focused on the progression of the initial damage around an indentation. They introduced a physically-based surface rolling contact fatigue (SRCF) model to predict material particle detachment, and used this model to describe the mechanism that controls damage propagation for large spalls (larger than the Hertzian contact width) [15]. In this model, the damage propagation is simulated by removing material elements that accumulate fatigue damage. However, this scheme is not specific to crack propagation, which causes flake release. Although the damage progression driven by the changing geometry of the contact and related cycling stresses was referred to, the crack propagation mechanism within the material was not addressed. Thus, that study focused on simulating the damage accumulation in the vicinity of the spall trailing edge until release of a fragment from the raceway.

**Figure 1:**
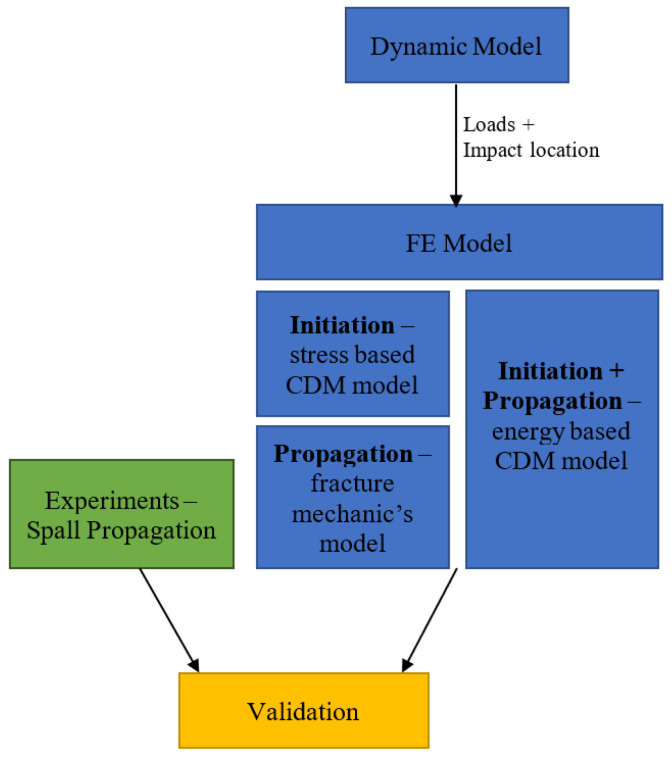
Research flowchart structure.

Understanding spall propagation involves three branches of knowledge: the dynamics of the impact by the rolling elements, the resultant mechanical fields in the race material, and the evolution of damage and cracks. Different damage models enable the different stages of spall propagation to be analyzed. Two damage mechanics models were embedded separately into the finite element (FE) model to simulate initiation and propagation of cracks. An alternative approach to the crack propagation stage was done in the framework of fracture mechanics. It required examination of the stress state at the crack tips in the vicinity of the spall edge. The proposed spall propagation mechanism may have the ability to predict the number of cycles until the fragment disconnects from the raceway and may eventually lead to development of a RUL model. The simulation results were supported and validated by experimental observations of several spalls by scanning electron microscopy (SEM), optical microscopy (OM), and micro-computerized tomography (CT) (Figure 1).

This article is organized as follows: Section 2 presents the methodology, which includes tools and models used for the proposed spall propagation mechanism. In Section 3, the experimental results (Section 3.1) and simulation results (Section 3.2 and Section 3.3) are presented. In this section, we describe the crack initiation (surface and sub-surface) process at the spall edge using effective stress CDM models (Section 3.2) and the crack propagation process using two approaches (Section 3.3). In addition, the effect of impact location on the crack propagation is also described in Section 3.3.1. The two proposed approaches for the crack propagation process are: (1) a fracture mechanics approach, described in Section 3.3.2 and (2) an accumulated inelastic hysteresis energy CDM approach, described in Section 3.3.3.

## 2. Methodology

### 2.1. Theoretical Methods

The particle release mechanism from the spall edge was analyzed in this work by a model for crack initiation and propagation in front of the spall and beneath its surface, until a particle is released. This section presents different physics-based models and tools that examine the mechanism governing spall propagation. These include: (a) a FE model that simulates the impact between a single rolling element (RE) and the spall edge, (b) the impact location between the RE and the spall edge and the contact force, taken from the dynamic model (used as boundary conditions for the FE model); (c) a damage model embedded into the FE model in order to examine crack initiation and propagation at the spall edge, and activated after the creation of plastic deformation and residual stresses within the spall edge; and (d) fracture mechanics applied to describe the crack propagation.

#### 2.1.1. Dynamic Model

A validated dynamic model [16] was utilized to compute the contact force between the RE and spall edge during impact, $F_{n}$, as well as the RE–spall impact location $\left(x_{imp},y_{imp}\right)$. The model is based on classic kinematics and dynamic equations. The boundary conditions for the dynamic model were defined according to the endurance experimental setup. More details about the dynamic model can be found in [16,17].

#### 2.1.2. FE Model

**Figure 2:**
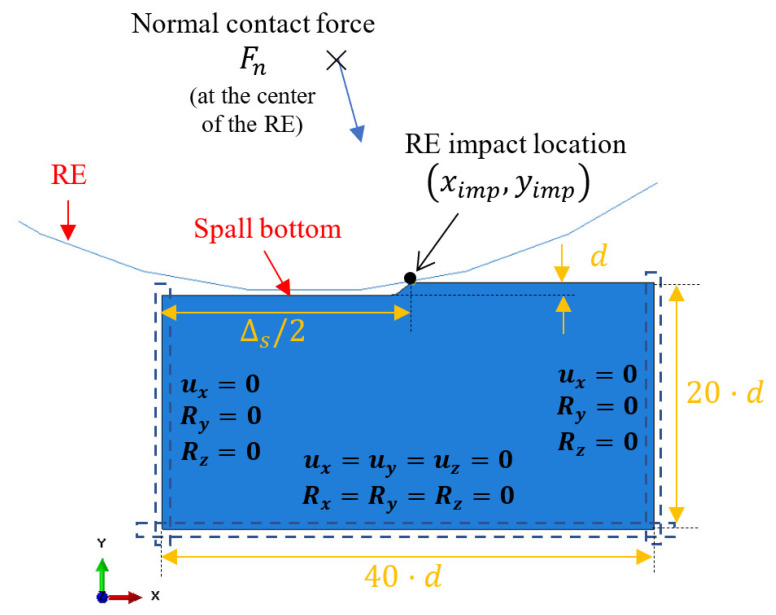
Geometry and boundary conditions of the FE model. The parameters values are listed in Table 1.

In order to understand the mechanics governing the fragment release from the spall edge, a FE model was developed based on the model in [17]. The model simulates the impact between a single RE and the spall edge. The normal contact force, $F_{n}$, at the time of impact and the impact location $\left(x_{imp},y_{imp}\right)$ were calculated by the dynamic model and used as boundary conditions in the FE model. The model geometry, boundary conditions, and material properties of the spall edge were determined according to [17] and are illustrated and listed in Figure 2 and Table 1. Several damage models (described in Section 2.1.3) and fracture mechanics tools (described in Section 2.1.4) were integrated into the FE model to examine the path of the cracks in the vicinity of the spall and compared with the experimental observations (presented in Section 3.1). For more details, see [18].

#### 2.1.3. CDM Model

Continuum damage mechanics (CDM) refers to various damaging processes in materials and structures at a macroscopic continuum level [19,20]. The damage model represents the microstructural damaged state and the deterioration of the material by a non-dimensional mechanical variable, D, which is accumulated due to fatigue loading. Regarding the damage variable, the constitutive equation takes the form:

(1) $$\tilde{\sigma }=E\left(1-D\right)\varepsilon$$

where $\tilde{\sigma }$ is the stress tensor after damage, E is the elastic modulus, and ε is the strain tensor. In [17], the fatigue damage is implemented by updating the effective elastic modulus, $\tilde{E}$, in the presence of damage by:

(2) $$\tilde{E}=E\left(1-D\right)$$

As can be seen from Equations (1) and (2), the damage affects the stiffness of the material. The damage variable, D, takes values ranging from 0, which represents undamaged material, to 1, which represents material that is completely damaged. However, to avoid numerical errors, a limit was set to the maximum value, $D_{max}$, in the FE model:

(3) $$0\leq D\leq D_{max}.$$

When damage at some point reaches its critical value, $D_{max}$, it corresponds to the initiation of a micro-crack. In this work, $D_{max}=0.9$ for the effective stress CDM model and $D_{max}=1$ for the accumulated inelastic hysteresis energy CDM model, and each element that reached this value lost its load bearing capability either for the initiation or the propagation stages.

The step-by-step description of the fragment release mechanism within the spall edge is modeled using CDM models. The description of crack initiation is based on an effective stress CDM model, and the crack propagation and overall process of fragment release are based on accumulated inelastic hysteresis energy.

##### CDM Model–Effective Stress

The CDM model, based on effective stress [21,22,23,24], enables information to be obtained about the initiation stage of cracks in the vicinity of a spall. The damage rate of evolution is given by:

(4) $$\frac{dD}{dN}={\left[\frac{\sigma_{eff}}{\sigma_{r}\left(1-D\right)}\right]}^{m}$$

where N is the number of stress cycles, $\sigma_{eff}$ is the effective stress that causes the damage, and $\sigma_{r}$ and m are material-dependent parameters. More about the damage algorithm and its application can be found in [17,25]. The spall propagation mechanism is divided into several stages where, in each stage, cracks initiate at different locations at the spall edge. In each stage, different stresses were used as the cause of the damage with the CDM modeling approach.

##### CDM Model–Accumulated Inelastic Hysteresis Energy

The proposed model, which simulates a single fragment formation, uses a CDM model that is based on the accumulation of plastic strain. In this model, the material damage initiation and evolution criteria depend on the stabilized accumulated inelastic hysteresis energy per cycle, Δw, [26,27,28].

The damage initiation criterion assumes a number of cycles, $N_{0}$, in which the degradation of the material response is initiated and is given by:

(5) $$N_{0}=c_{1}\Delta w^{c_{2}}$$

where $c_{1}$ and $c_{2}$ are material constants. After N cycles, in which the structure reaches stability, for each material point in the structure, the accumulated inelastic hysteresis energy per cycle, Δw, is calculated, and the number of cycles of N is compared to $N_{0}$. If the criterion $N>N_{0}$ is satisfied at any material point, the damage variable, D, is calculated and updated based on a damage evolution criterion. The damage evolution criterion is given by:

(6) $$\frac{dD}{dN}=\frac{c_{3}\Delta w^{c_{4}}}{L}$$

where $c_{3}$ and $c_{4}$ are material constants, and L is the characteristic length associated with an integration point.

In order to avoid extensive computational time, a low-cycle fatigue analysis is accelerated, and each increment extrapolates the current damaged variable in the bulk material forward over many cycles to a new damaged variable after the current loading cycle is stabilized. The material constants $c_{1}$, $c_{2}$, $c_{3}$ and $c_{4}$ for the simulation are listed in Table 2 and chosen based on [27].

#### 2.1.4. Fracture Mechanics

Once the initial cracks started to evolve in the simulation, using CDM models, real cracks were implemented at the same location using tools for fracture mechanics in ABAQUS. Fracture mechanics was used to select the path of the propagating cracks. At each step, the stress distribution, especially at the crack tip, was calculated. The crack propagation direction (CPD) was predicted by examining three different built-in criteria: (a) maximum tangential stress, (b) maximum energy release rate, and (c) $K_{II}=0$. These criteria yielded similar predictions with a maximum error of 6%. The criterion of $K_{II}=0$ was selected for the rest of the investigation. All three CPD criteria predict the angle at which a pre-existing crack propagates for each one of the specified conditions (a–c). In addition, the maximum principal stress vectors (orientation and magnitude) at the crack tip were also considered.

### 2.2. Experimental Methods

Two types of bearings were tested: the Sikorsky CH-53 helicopter swashplate bearing and an6206 ETN9. For the latter, a 0.75 mm bore diameter was initially seeded on the outer race in order to accelerate the bearing deterioration.

**Figure 3:**
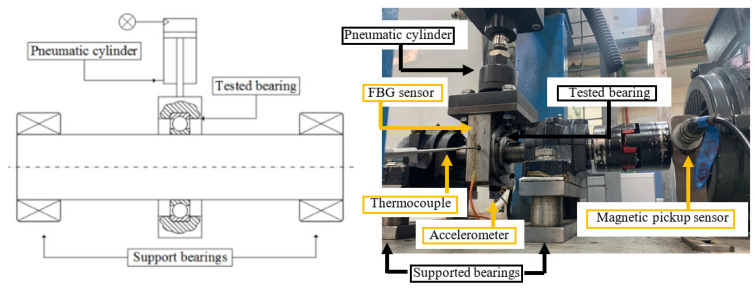
A schematic drawing and a picture of the test rig.

The experimental setup is illustrated in Figure 3. The pneumatic cylinder applied vertical load of 2200 N on the tested bearing, and the rotational speed of the shaft was 35 Hz. Additional details on the experimental setup and results are presented in [18]. Here, we present only relevant observations to the current research.

After each experiment, the bearings were inspected by stereomicroscope. They were sectioned, and the bearing surfaces were inspected with a scanning electron microscope (SEM). Selected areas were then sectioned, mounted, polished up to a 0.3 μm level, and inspected with an optical microscope. Some bearings were inspected by RX Solution^TM^ micro-CT.

## 3. Results

### 3.1. Experimental Results

**Figure 4:**
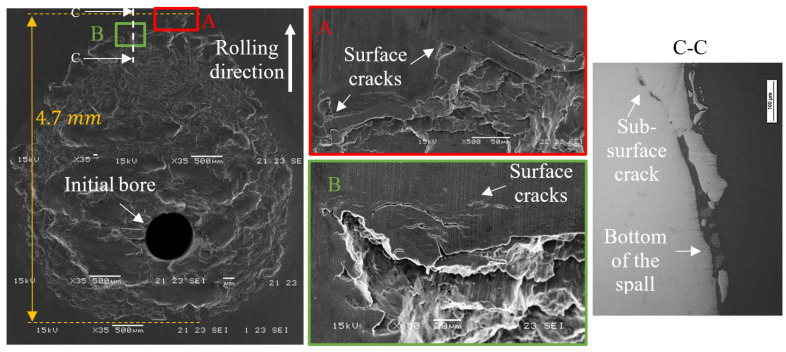
SEM images of a spall that was initiated by a bore. Surface cracks appear in front of the spall (**A**,**B**), and sub-surface cracks appear underneath the trailing edge (**C**).

SEM images of a spall are illustrated in Figure 4. Several surface and sub-surface cracks appeared at its trailing edge. The first two cracks, a surface and sub-surface crack that propagate and connect to each other, released the fragment. Based on Figure 4, it is difficult to determine which type of crack initiated the drop of the first fragment; however, evidence of three types of cracks were revealed: (i) sub-surface cracks that propagated horizontally before surface trailing edge cracks propagated vertically, (ii) a single sub-surface crack that propagated and reached the surface, and (iii) the reverse of the first type—surface cracks that propagated vertically before sub-surface cracks propagated horizontally. In the first and third types of fragment release, the cracks may initiate simultaneously; however, sub-surface and surface cracks are more dominant, respectively. It is noteworthy to mention that all the experimental analyses of the spalls showed similar results, and also are supported by [14,18,29,30].

**Figure 5:**
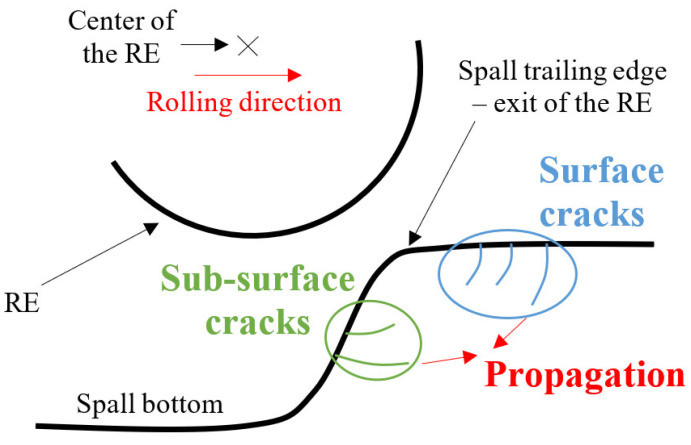
Schematic description of the fragment release from the spall edge, including the initiation of surface and sub-surface cracks. The cracks propagate until a fragment is released from the raceway.

Based on the experimental observations, the spall propagation process can be described as schematically presented in Figure 5. Fragment detachment from the spall occurred as a result of the appearance of: (i) sub-surface cracks underneath the spall trailing edge, (ii) surface cracks in front of the trailing edge of the spall, and (iii) crack propagation until a fragment was released from the raceway.

### 3.2. Simulations of Crack Initiation at Spall Edge

In order to better understand the mechanics governing crack initiation at the spall edge (surface and sub-surface cracks), two models were developed based on the tools and physics-based models presented in Section 2 and on the experimental results.

Section 3.2.1 describes the initiation of sub-surface cracks that appeared underneath the trailing edge of the spall, and Section 3.2.2 describes the initiation of surface cracks that appeared at the trailing edge of the spall—both based on an effective stress CDM model. The two models have two steps: (1) pre-loading calculated as a contact problem, and (2) applying the damage model on the FE model (more details can be found in [17]).

#### 3.2.1. Sub-Surface Cracks

**Figure 6:**
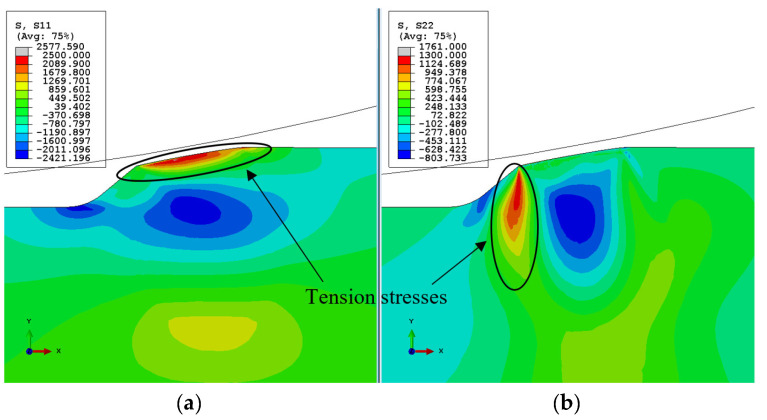
Simulated residual stresses (**a**) in the horizontal direction x, and (**b**) in the vertical direction y, in the unloading stage.

**Figure 7:**
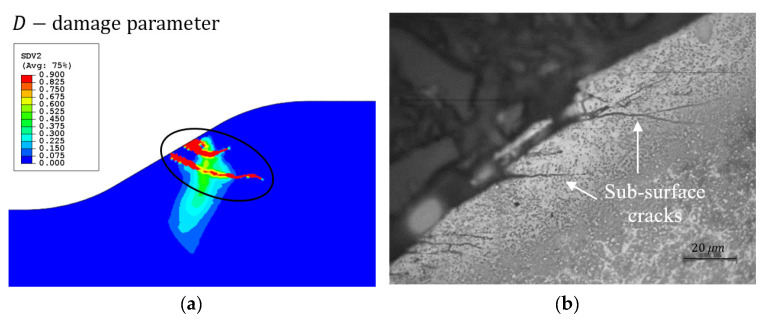
(**a**) Map of the damage variable, D, at the end of the crack initiation simulation, representing the crack generation beneath the spall edge surface, where $S_{22}$ is used as the effective stress in Equation (4). In the simulation of the crack propagation, only the initial part of the crack was taken. (**b**) Microscopic image of a metallographic cross-section through a spall front, etched with nital.

Using an effective stress CDM model, Equation (4) enables the investigation of the initiation of both surface and sub-surface cracks, as presented in Figure 5. It was assumed that for each type of crack, a different stress component controls the crack initiation; therefore, only this component was included in the damage model. A tensile residual stress $S_{11}$ appears in front of the spall (Figure 6a), and tensile residual stress $S_{22}$ appears in the region of the free edge of the spall (Figure 6b). Similar distributions of residual stresses under rolling bearing surfaces were found in several theoretical works [10,11,12,17]. This implies the possibility of vertical crack propagation at the trailing edge of the spall and horizontal cracks from the sub-surface of the spall. To simulate the sub-surface cracks, the effective stress, $\sigma_{eff}$ (Equation (4)), is assumed to be the vertical stress, $S_{22}$. In Figure 7a, the CDM damage simulation results are presented. Two cracks were generated beneath the spall’s trailing edge surface. Experimental observations of the spall edge showed cracks in the sub-surface of the spall edge. In the close-up image of the cross-section observed by light microscope (Figure 7b), several cracks were generated beneath the surface in the horizontal direction, which supports the simulation results.

#### 3.2.2. Surface Cracks

**Figure 8:**
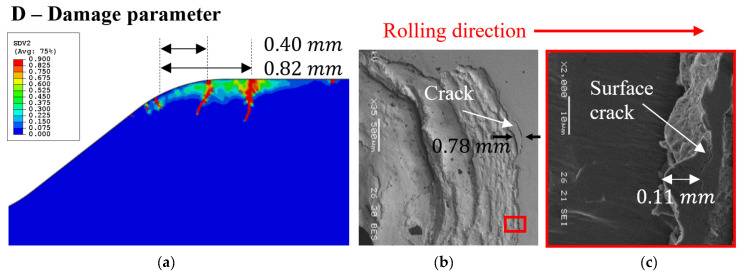
(**a**) Map of the damage variable, D, represents the crack generation within the spall edge, where the maximum residual stress uses as the effective stress. (**b**) SEM images of the spall edge with surface cracks in front of the spall. (**c**) Close-up image of the front of the spall.

The effective stress CDM model was also used to study the propagation of the surface cracks. For these cracks, $S_{11}$ was assumed as the effective stress, $\sigma_{eff}$, in Equation (4) due to the fact that $S_{11}$ represents tensile stresses in front of the spall (see Figure 6a), and when the RE impacts the spall edge, those stresses become compressive. These cyclic stresses, superposition of residual stresses and the applied loading, promote the formation of fatigue cracks in front of the spall, perpendicular to the free surface. The maximum residual stress CDM damage simulation results are presented in Figure 8a. The cracks initiated at the surface of the spall were similar in shape and location to those observed by SEM in the tested bearings presented in Section 3.1, in Figure 8b,c, and in the experimental observations in [11,12,31].

### 3.3. Simulations of Crack Propagation

#### 3.3.1. The Effect of Impact Location

**Figure 9:**
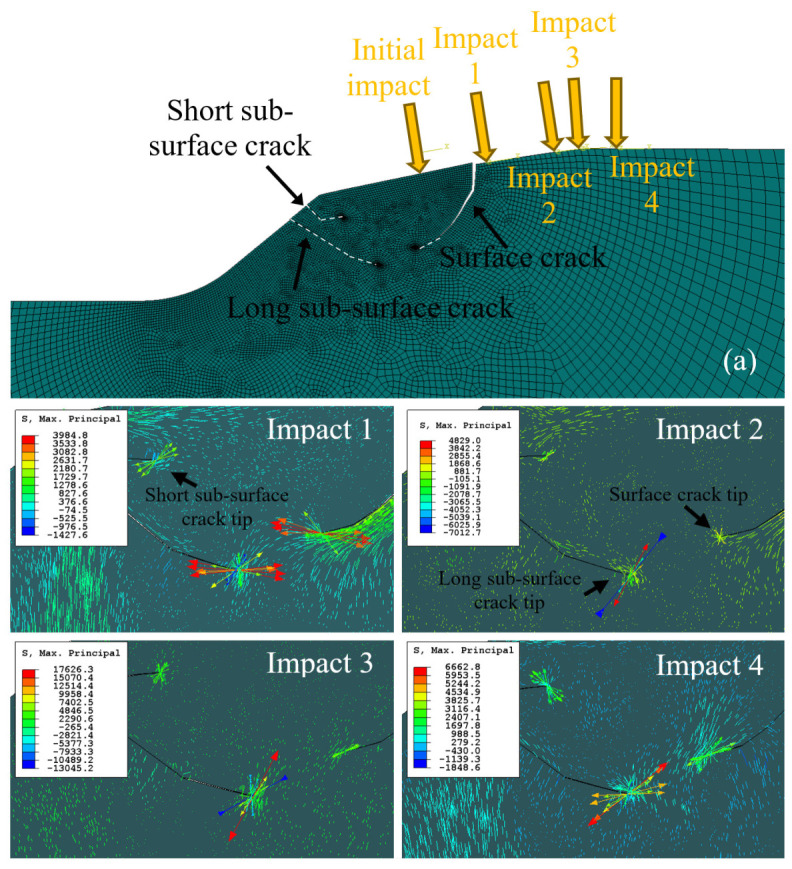
Residual maximum principal stress direction and magnitude following an RE impact at four locations. The impact locations are indicated in Figure (**a**). The crack tips are distinguished by the finer mesh. Red arrows indicate tensile stresses, and blue arrows indicate compressive stresses.

In this section, we study the propagation of the two types of cracks initiated in the previous section. The propagation is controlled by the stresses generated by the impact of the REs. The RE–spall edge interaction has two characteristics: (a) after the first collision of the RE on the spall’s edge, the RE rattles on the raceways several times until it is locked back between the raceways [32,33,34], and (b) the impact location of the RE changes with every hit of the RE on the spall edge [35]. It is assumed that the different impact locations and the rattling phenomenon can affect the crack propagation process. To examine this process, the impact location, $\left(x_{imp},y_{imp}\right)$, was varied, keeping the same normal contact load, $F_{n}$, as calculated in the dynamic model. These impacts produce plastic compressive deformation that turn into residual tension (Figure 6 and see [18]). For every different impact location, the residual maximum principal stress direction was extracted, and their direction and amplitude are shown in Figure 9. The maximum principal stresses at the tips of the sub-surface cracks and the surface crack were affected by the location of the impact. For example, in Impact 2, the residual maximum principal stresses at the surface and sub-surface crack tips were compressive. In Impact 3, the residual stress only on the sub-surface crack was tensile, and in Impacts 1 and 4, the two crack tips remained under tensile stress. In parallel, the short sub-surface crack was also in a tensile condition following Impacts 1 and 4, and it could propagate toward the surface of the spall edge until a small fragment is released. We assume that the residual tension is the driving force for the propagation of the cracks. It seems that, at every impact location, different plastic zones are generated, and the crack propagation switches between the surface crack and the long sub-surface crack. Thus, the crack paths are expected to vary with the history of impact locations by the Res. Eventually, the two cracks will coalesce and release a fragment. Because prediction of the crack paths is a complex task, we restrict this discussion to a single impact location (marked as “Initial impact” in Figure 9a), but use two approaches in order to evaluate their conformance with the experiment: (1) a fracture mechanics approach, described in Section 3.3.2, and (2) an accumulated inelastic hysteresis energy CDM approach, described in Section 3.3.3.

#### 3.3.2. Fracture Mechanics Approach

**Figure 10:**
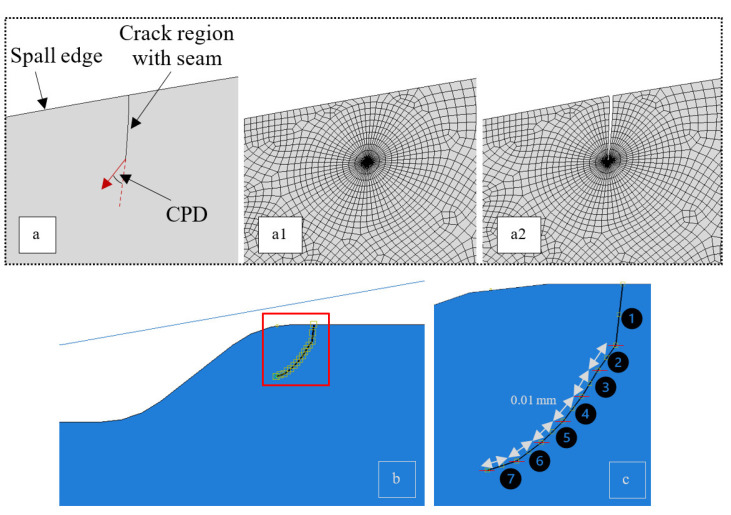
Modeling of a crack. (**a**) Example of a seam embedded in the initial surface crack at the spall edge. (**a1**) Before assigning the “seam”; (**a2**) after assigning the “seam”. (**b**) Seam embedded in the surface crack at the spall edge. (**c**) Close-up image of the crack. The crack is represented by seven segments; each segment from 2 to 7 has a length of 0.01 mm.

The initial damage locations were chosen according to the damage simulation results presented in Section 3.2.1 and Section 3.2.2, and are taken to represent initial cracks. The crack propagation process is studied here, applying notions of fracture mechanics. To model the cracks in the vicinity of the spall, a “seam” of overlapping duplicate nodes was assigned to the initial crack regions when the mesh was generated [28]. An example of a seam embedded in the initial surface crack at the spall edge is presented in Figure 10a. A contact problem was defined between the crack faces—“hard” normal behavior with a friction coefficient of 0.8. The simulation proceeded in cycles, each consisting of three steps: (1) loading and unloading of the RE on the spall edge, (2) crack opening, and (3) re-loading and unloading of the RE on the spall edge to obtain the updated stress fields. From the simulation results, the stress intensity factor and the CPD were extracted according to Section 2.1.4. The CPD criterion predicts the angle at which a pre-existing crack propagates (Figure 10a). The crack was extended by 0.01 mm in each cycle. This process was repeated until the value of the stress intensity factor reached a small value, close to 0, which is interpreted as crack arrest. An example of surface crack propagation is presented in Figure 10b,c, where the crack is represented by seven segments.

**Figure 11:**
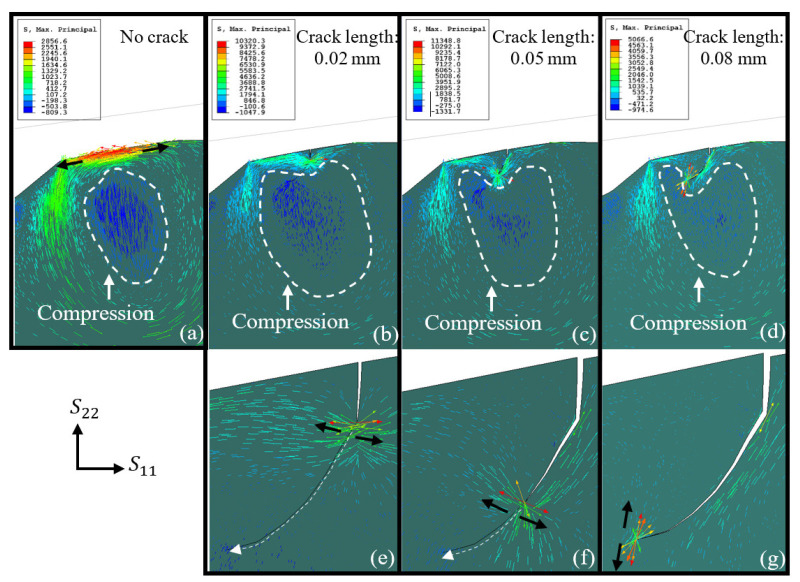
The residual maximum principal stress orientation and magnitude (Mpa) for the unloading stage where (**a**) no crack is implemented, (**b**) crack length of 0.02 mm, (**c**) crack length of 0.05 mm, and (**d**) crack length of 0.07 mm. (**e**–**g**) are close-ups of the surface cracks in (**b**–**d**), respectively.

**Figure 12:**
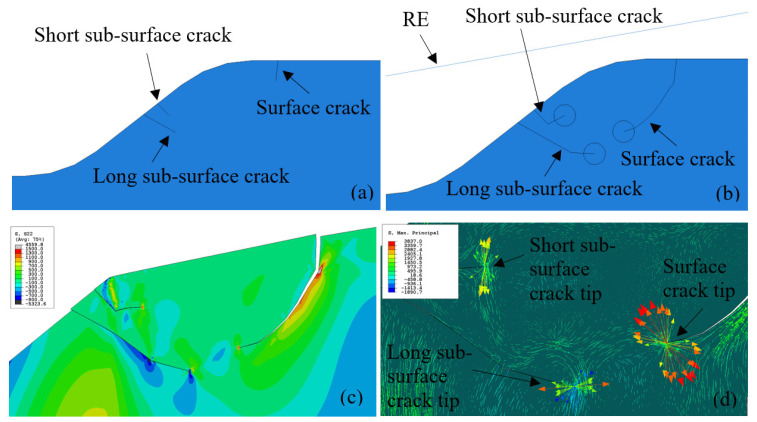
(**a**) Crack implementation in the vicinity of the spall in the initial configuration. Crack lengths: short sub-surface crack—0.02 mm, long sub-surface crack—0.06 mm, surface crack—0.02 mm. (**b**) Crack implementation in the vicinity of the spall in the final stage of propagation. Crack lengths: short sub-surface crack—0.04 mm, long sub-surface crack—0.08 mm, surface crack—0.08 mm. (**c**) $S_{22}$ stress field. (**d**) Close-up of the crack tip presents the residual maximum principal stress orientation and magnitude.

In Figure 11, the residual maximum principal stress is represented as vectors at every integration point, each corresponding to the principal value and orientation along the corresponding principal direction. After the pre-loading stage, tensile stresses appeared close to the trailing edge surface. In the pre-loading stage, when the RE impacts the spall edge, the tensile stresses become compressive stresses. The periodic behavior of the stresses creates an environment that can explain the progression of fatigue cracks on the surface of a spall. Beneath the tensile area, there is a compression area that can interfere with crack propagation. In the crack propagation process, crack lengths of 0.02 mm, 0.05 mm, and 0.08 mm (see Figure 11), it seems that the compression area withdraws for each crack growth increment since the crack releases the residual stresses, and the compression edge delineates the crack path. The maximum principal stress at the crack tip changes its direction, from a horizontal to vertical direction. It can be assumed that the criterion for surface crack propagation starts as a horizontal stress, $S_{11}$, used as the effective damage-propagation stress, and then changes to a vertical stress, $S_{22}$. The same process was performed for the sub-surface crack. Figure 12 presents an undeformed spall edge shape with multiple cracks (one surface crack and two sub-surface cracks) in the first stage of propagation (Figure 12a), and in the final stage of propagation (Figure 12b). The vertical stresses, $S_{22}$, after the loading and unloading stage are shown in Figure 12c. It seems that the long sub-surface crack tip is subjected to compression stresses and the surface crack tip to tension. In addition, the residual maximum principal stress direction was also examined and gave similar results (Figure 12d). The direction of the tensile residual principal stresses of the trailing edge surface crack was vertical to the crack direction, which caused the crack to grow; however, the residual maximum principal stresses of the long sub-surface crack caused the crack tip to cease. This condition indicates that the surface crack tends to propagate toward the long sub-surface crack until a fragment is released.

#### 3.3.3. CDM Approach

**Figure 13:**
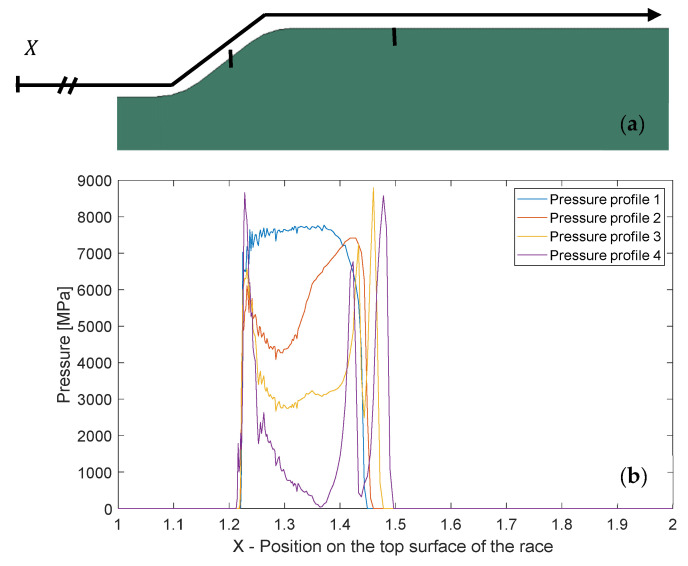
(**a**) Part of the spall edge from the model with axis X that represents the location on the race. (**b**) Four different pressure distributions produced from the simulation as a result of four different damage conditions (“Pressure profile 1” applies to the initial state; Profiles 2–4 apply to the damaged spall edge).

An alternative approach to simulate the overall fragment release process, damage initiation, and propagation is by using the accumulated inelastic hysteresis energy CDM model, Equations (5) and (6) in Section “*CDM Model–Accumulated Inelastic Hysteresis Energy*”, respectively. The boundary condition for the simulation is the normal impact force and location obtained from the dynamic model. Then, the simulation is composed of two steps, which are repeated until the damage propagates and forms the contour of a fragment. Step (1) consists of pre-loading, and solves by FEM the contact problem in which the RE is pressed against the spall edge. Then, the normal pressure distribution on the spall edge (as can be seen in Figure 13a) is extracted, and finally, the RE is unloaded from the raceway. In Step (2), cyclic loading is done with a direct cyclic approach in ABAQUS that uses as boundary conditions the pressure distribution from Step (1). In Figure 13b, four pressure distributions extracted from our simulation are presented. Profile 1 is the pressure distribution before damage evolved. The other profiles describe the evolution of the pressure with growing damage.

**Figure 14:**
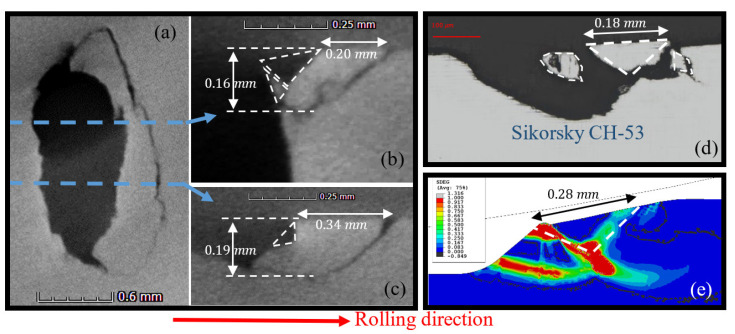
Comparison between the FE model and spall images: (**a**–**c**) Micro-CT of the spall trailing edge from the third experiment; (**b**,**c**) are cross-sections of image (**a**) in different locations. Several small fragments are detached or about to detach from the raceway (white dashed lines). (**d**) OM image of the swashplate bearing of a Sikorsky CH-53 helicopter. (**e**) Simulation results of the accumulated inelastic hysteresis energy CDM model.

A comparison between simulation and experimental results is presented in Figure 14. The simulation result in Figure 14e is compared with two sections taken from micro-CT imaging of spalls from endurance tests of bearings (Figure 14a–c) and to an OM image taken from a natural in-service spall that appeared in a Sikorsky CH-53 helicopter swashplate bearing (Figure 14d). In the two sections (Figure 14b,c), the same behavior was observed. The simulation results show small fragments that detached from the large fragment, which can also be observed in the CT and OM images (Figure 14a–d). This phenomenon creates a wavy appearance within the spall. That small fragments are released from the spall edge can also be supported by [36], who studied the damage severity of spalled bearings based on oil debris monitoring (ODM). The authors found that the particle size distribution during different stages of the spalling process remained relatively consistent, indicating that small fragments are always released from the raceway during bearing operation.

The details of the fragment release from the raceway may vary according to the different directions and locations of the impact, affecting the path of the crack propagation.

**Figure 15:**
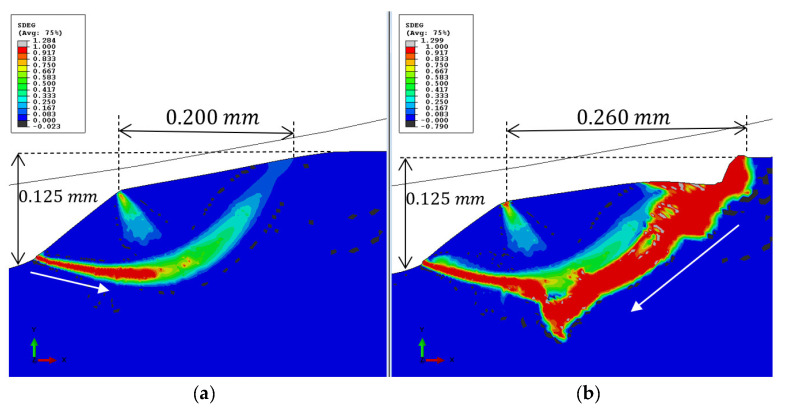
Simulation results of parameter D, using the accumulated inelastic hysteresis energy CDM model. (**a**) Presence of only a sub-surface crack at the spall edge, (**b**) appearance of surface cracks after changing the pressure distribution at the spall edge.

The simulation results satisfactorily predicted the size and shape of the fragments well. The fragment size and aspect ratio (shape) from the simulation (presented in Figure 15) were similar to those of the fragments observed in the images in Figure 14. In the experimental observations (Section 3.1), two types of phenomena were identified: (a) the surface and sub-surface cracks initiated consecutively one before another, or they may have initiated simultaneously. (b) A fragment may be released due to the presence of only sub-surface cracks (without trailing edge cracks). Figure 15 illustrates a typical simulation of spall damage propagation. A sub-surface crack initiation is depicted in Figure 15a. If the pressure profile is sustained as Profile 1 in Figure 13b, the sub-surface crack propagates to the opposite free surface and generates a fragment. If the pressure profile is updated with the evolving damage through Profiles 2–4, damage is initiated on the trailing edge surface and advances towards the sub-surface crack, as shown in Figure 15b. In most of the simulations, sub-surface cracks were generated before surface cracks, and the cracks propagated and coalesced. Finding the parameters of a damage model that corresponds to the tested bearing can help in building a prognostic tool.

## 4. Conclusions

Previous work dealt with spall initiation during rolling contact fatigue and stress analysis in the vicinity of defects near the surface [10,11,12,37], whereas, spall propagation was the focus of the present work. The process in bearing raceways was divided into three stages: the appearance of surface and sub-surface cracks, followed by their propagation, and ending with the release of a fragment from the raceway. In every stage, several cracks form simultaneously. The coalescence of surface and sub-surface cracks or of at sub-surface crack reaching the surface cause fragment release. Evidence of cracks can be seen in the experimental observations. All three stages were examined using CDM models and/or fracture mechanics. The formation of cracks in the vicinity of a spall (surface and sub-surface cracks) was carefully studied using the effective stress CDM model, and the propagation of those cracks was examined using fracture mechanics tools and the accumulated inelastic hysteresis energy CDM model. A proposed model that predicts the overall process of a single fragment release also integrates the accumulated inelastic hysteresis energy CDM model.

- A dynamic model was used to determine the stresses due to RE impact events. The impact location of the RE on the spall edge has great influence on the crack behavior in terms of their appearance timing and location. Different fragment shapes and sizes results.
- The residual stresses caused by the RE–spall edge impact are tensile in front of the spall, and underneath it, the stresses are compressive, which prevent crack propagation. The appearance of surface and sub-surface cracks releases the residual stresses and changes areas of compression stresses into tension, causing crack propagation until a fragment is released.
- Integrating a dynamic model that can simulate different spall sizes in the FE spall propagation model establishes a constitutive law of spall deterioration.

Future work will study the material parameters for the proposed overall spall propagation model. This model will serve as a foundation for developing a physics-based prognostic tool for ball bearings.

## Figures and Tables

**Table 1:** FE model parameters and material properties (for M50 steel).

Parameters	Value	Units
Spall depth—d	0.125	mm
Spall size—$\Delta_{s}$	2.6	mm
Impact location $\left(x_{imp},y_{imp}\right)$	$\left(1.3,\;2.1\times 10^{-3}\right)$	mm
Normal contact load—$F_{n}$	1900	N
Ball radius—$R_{RE}$	6.4	mm
Modulus of elasticity for spall edge—E	200	MPa
Poisson’s ratio for spall edge—υ	0.3	–
Plasticity property (assuming bilinear behavior) [12]	$\left(0.016,\;3080\right)$ $\left(0.300,\;3200\right)$	$\left(\varepsilon ,\;MPa\right)$

**Table 2:** Material constants for the CDM model based on accumulated inelastic hysteresis energy.

$c_{1}$	$c_{2}$	$c_{3}$	$c_{4}$
50	−0.9	2 × 10^−4^	1.15

## Data Availability

Not applicable.
